# Le syndrome miroir: revue de la littérature illustrée par un cas

**DOI:** 10.11604/pamj.2020.37.125.19337

**Published:** 2020-10-05

**Authors:** Khadija Benchekroune, Jihad Drissi, Mounir Moukit, Jaouad Kouach, Driss Moussaoui

**Affiliations:** 1 Service de Gynécologie-Obstétrique, Hôpital Militaire d´Instruction Mohamed V, Rabat, Maroc

**Keywords:** Syndrome de Ballantyne, anasarque, prééclampsie, Ballantyne syndrome, anasarca, pre-eclampsia

## Abstract

Le syndrome miroir ou syndrome de Ballantyne est une entité pathologique rare, définit par l´association d´une anasarque fœtale et d´un œdème maternel généralisé pouvant se compliquer d´une prééclampsie. L´objectif de ce travail est de mettre en exergue les particularités diagnostic et thérapeutique de cette entité clinique grave, qui, en dépit de sa rareté, ne doit pas être méconnu. Et ceux à travers la description d´un cas clinique d´une parturiente de 35 ans qui s´est présentée aux urgences, à 26 semaines d´aménorrhée, dans un tableau de syndrome miroir idiopathique dont l´évolution fut rapidement marquée par la survenue d´un décès in utéro puis régression de la symptomatologie clinico-biologique maternelle une fois la vacuité utérine obtenue. En effet, un traitement spécifique in utéro est vivement souhaitable. Quoique dans certains cas graves idiopathiques, seule l´obtention de la vacuité utérine permet l´amélioration du pronostic maternel voire fœtale.

## Abstract (en)

Mirror syndrome or Ballantyne syndrome is a rare disease, characterized by the association between fetal anasarca and maternal generalized edema. This may be complicated by pre-eclampsia. The purpose of this study was to highlight the diagnostic and therapeutic features of this severe clinical entity, which, despite its rarity, should be suspected. We here report the clinical case of a 35-year-old parturient woman at 26 weeks of amenorrhea presenting to the Emergency Department with idiopathic mirror syndrome whose progression was rapidly marked by the occurrence of intrauterine fetal death and remission of maternal clinical-biological symptoms, once uterine vacuity was obtained. Indeed, a specific treatment in uterus is necessary, although in some severe idiopathic cases, only uterine vacuity can improve maternal and fetal prognosis.

## Introduction

Le syndrome miroir, syndrome de Ballantyne ou triple œdème, est une entité pathologique rare, décrite pour la première fois en 1892 par JW Ballantyne, elle se définit par l´association d´une anasarque fœtale immune ou non immune, et d´un œdème maternel généralisé pouvant se compliquer d´une prééclampsie [1]. L´objectif de ce travail est de mettre en exergue les particularités diagnostic et thérapeutique de cette entité clinique grave, qui, en dépit de sa rareté, ne doit pas être méconnu.

## Patient et observation

**Figure 1:**
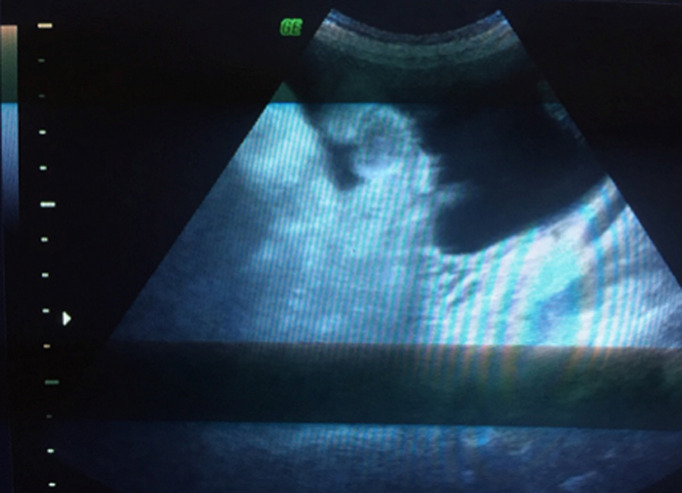
ascite maternelle (anses grêliques flottantes)

**Figure 2:**
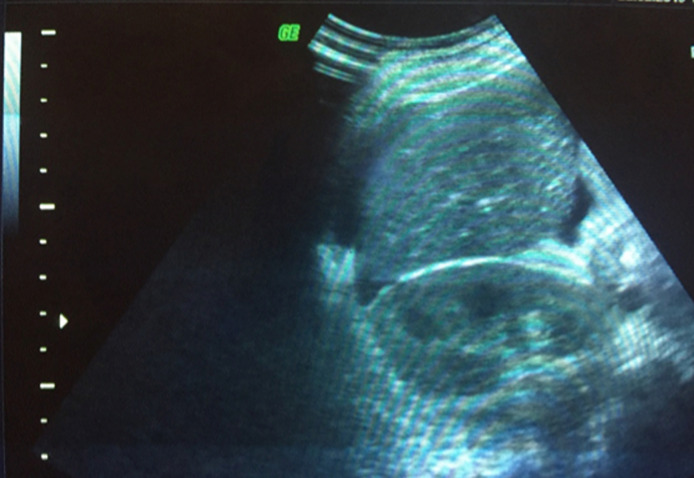
espace de Morrison; ascite de moyenne abondance

**Figure 3:**
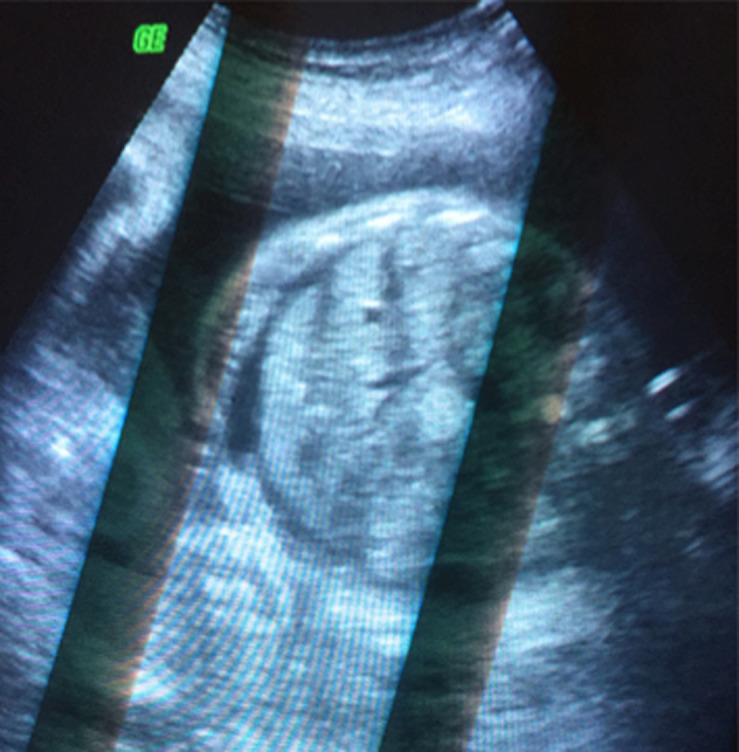
ascite fœtale

**Figure 4:**
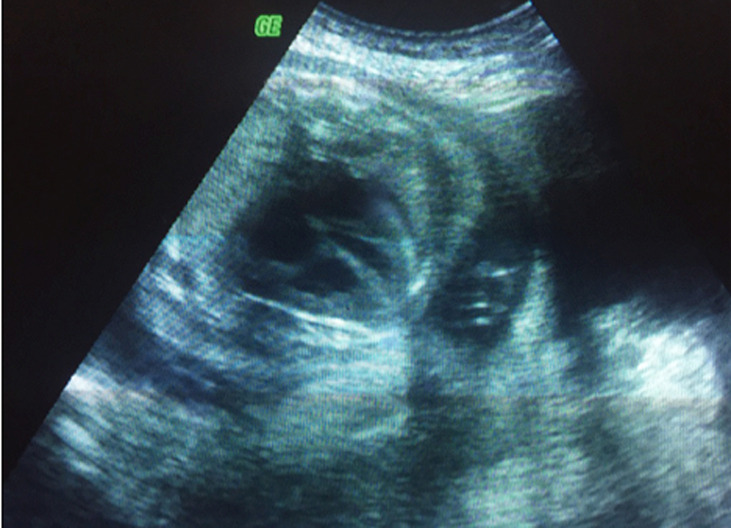
épanchement péricardique fœtale

Il s´agit d´une patiente de 35 ans, de groupe sanguin O Rhésus positif, troisième geste, deuxième part, mère de deux enfants. Sans antécédents pathologiques notables. Admise à 26 semaines d´aménorrhée dans un tableau de prééclampsie sévère: chiffres tensionnels variant entre 140 et 150mmhg de systolique, 80 et 90mmHg de diastolique sous traitement médical à base d´alpha méthyl dopa 1500mg/j et une protéinurie à 5g/24h. Le reste du bilan biologique vasculorénal était par ailleurs normal. L´examen obstétrical avait objectivé une hauteur utérine correspondant à l´âge gestationnel, avec à la palpation de l´abdomen une matité déclive en faveur d´une ascite maternelle confirmée à l´échographie abdominale (Figure 1, Figure 2). L´échographie obstétricale est revenue en faveur d´une grossesse monofœtale évolutive avec une biométrie correspondant à l´âge gestationnel et un anasarque fœtale: ascite, pleurésie et discret épanchement péricardique, sans épaississement placentaire et sans anomalies morphologiques décelables (Figure 3, Figure 4).

**Figure 5:**
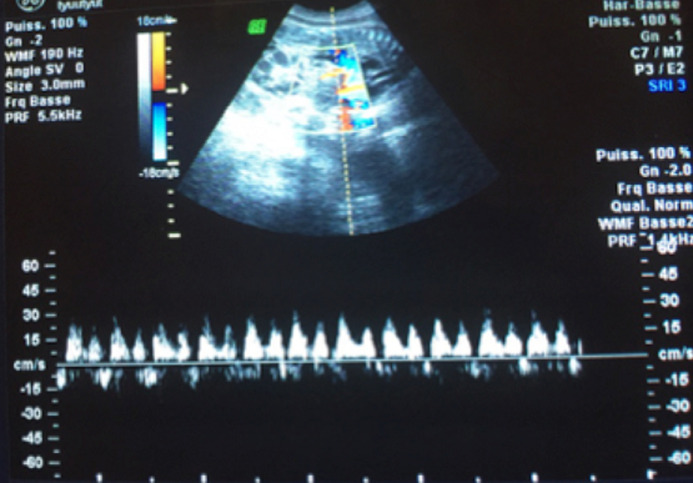
doppler du ductus venosus objectivant une onde à négative

Le doppler pulsé de l´artère cérébral moyenne objective un pic systolique de vélocimétrie à 32cm/s (<1.5 MOM) ce qui n´était pas en faveur d´une anémie fœtale. Le doppler du ductus venosus avait par contre montré une onde à négative (Figure 5). Compte tenu de la stabilité de l´état maternel, l´âge jeune de la grossesse et les risques de la très grande prématurité, l´indication de l´extraction fœtal n´a pas été portée et la patiente a été gardée sous surveillance de ses paramètres cliniques et biologiques. L´évolution était marquée par la survenue d´une mort fœtale in utéro dans les 24 heures. Par ailleurs, la patiente est demeurée stable sur le plan clinique et biologique. Une maturation cervicale par analogues de prostaglandines a été instaurée ce qui a permis l´expulsion d´un mort-né de sexe féminin pesant 850g sans malformations identifiées. L´évolution maternelle était favorable avec maintien d´un bon équilibre tensionnel après dégression des doses de l´antihypertenseur, un bilan vasculorénal est prévu. Dans cette observation aucune étiologie évidente n´a été retrouvée.

## Discussion

Décrit pour la première fois en 1892 par John W Ballantyne, le syndrome miroir, également appelé syndrome de Ballantyne ou encore triple œdème, est une entité pathologique rare qui compliquerait 50% des hydrops fœtalis soit 1/6000 grossesse [2-4]. Il se définit par l´association d´une anasarque fœto-placentaire à un tableau d´œdème maternel généralisé avec hémodilution pouvant se compliquer d´un tableau pseudo-toxémique dans 50% des cas [1, 2]. D´autres signes peuvent s´observer de manière inconstante: sur le plan clinique, une élévation des chiffres tensionnels peut être retrouvé, comme cela a été le cas chez notre patiente. De même des céphalées, des troubles visuels ou une oligurie peuvent se rencontrer. Sur le plan biologique on peut assister à une élévation de l´uricémie, une anémie par hémodilution, une perturbation de la fonction rénale, une hypoprotidémie, une protéinurie faiblement élevée, une élévation des transaminases, et des désordres électrolytiques associant hyperkaliémie et hyponatrémie. Le taux de plaquette est normal ce qui permet de distinguer le syndrome miroir du Hellp syndrome [1, 2, 5]. Aussi faut-il souligner que l´hémodilution, principal signe biologique retrouvée dans 89% des cas, contraste avec l´hémoconcentration classiquement retrouvée en cas de prééclampsie [4]. L´âge gestationnel de diagnostic est très variable, les cas décrits dans la littérature ont été diagnostiqués entre 16 et 34 semaines d´aménorrhée (SA) [1, 6].

Dans notre observation le diagnostic fut posé à 26SA. Le premier cas rapporté dans la littérature était associé à une anasarque fœtale immune, depuis, plus de 57 cas ont été rapportés avec des étiologies très variées. En effet, en 2000, Medley et Harding ont décrit un cas en rapport avec une tachycardie supraventriculaire à 240bpm à 27SA et dont l´évolution était spectaculaire sous antiarythmiques (Flécaine) [4, 7]. La disparition du syndrome miroir a également été rapportée après fœticide d´un jumeau présentant une anasarque inexpliquée à 16SA ce qui a permis la naissance à terme de l´autre jumeau. Touhamy *et al*. ont également rapportés le cas d´un syndrome de Ballantyne dû à une malformation cardiaque complexe. Ceci dit, toute étiologie immune ou non immune pouvant aboutir à l´apparition d´une anasarque fœtale sévère peut se compliquer d´un syndrome miroir [2]. Le mécanisme physiopathologique reste mal élucidé. Malgré les similitudes cliniques avec la prééclampsie les mécanismes placentaires semblent différents. Alors que le défaut d´invasion trophoblastique est le primum movens de l´ischémie placentaire à l´origine de la prééclampsie, l´ischémie placentaire observée en cas de syndrome miroir est secondaire à hypovascularisation consécutive à l´œdème placentaire [1, 4]. Il n´est donc pas étonnant de retrouver une augmentation des facteurs antiangiogéniques tel que le récepteur soluble du facteur de croissance de l´endothélium vasculaire (sVEGF R-1) et le soluble Fms-like tyrosine kinase et une diminution des facteurs angiogéniques tel que le facteur de croissance placentaire (PIGF).

Cette ischémie placentaire est associée à la libération de radicaux libres, lipides oxydés, cytokines et leptines avec augmentation de la perméabilité vasculaire, une vasoconstriction et une activation des systèmes de la coagulation intravasculaire à l´origine des manifestations clinico-biologiques du syndrome miroir. Le pronostic fœtal est le plus souvent réservé avec la survenu très fréquente de décès in utéro en rapport avec la sévérité de l´étiologie de l´anasarque [1, 8, 9]. Cependant, c´est le retentissement potentiellement morbide maternel qui en fait la particularité. Morbidité qui peut aller jusqu´au décès maternel par œdème aigu du poumon, insuffisance rénale ou crise convulsive [1]. La prise en charge dépend de l´état maternel, l´âge gestationnel, de l´étiologie identifiée et des possibilités de prise en charge in utéro. Un traitement spécifique in utéro est vivement souhaitable, il permet l´amélioration du pronostic fœtal, l´éviction de la très grande prématurité induite et la résolution des signes clinico-biologiques maternels. Dans tous les cas où il n´existe pas de cause curable identifiée et que le pronostic vital maternel est menacé, une interruption médicale de la grossesse doit être envisagée. Il doit permettre la normalisation des paramètres clinico-biologiques maternels aussitôt la vacuité utérine obtenue [2]. Dans certains cas on peut assister à une résolution spontanée de ce syndrome, cela a été décrit dans le cadre d´infections à parvovirus B19 [2]. Dans notre cas l´état maternel était stable, aucune étiologie n´a été identifiée, l´âge gestationnel était associé aux risques de la très grande prématurité, l´expectative a ainsi été de mise et l´évolution était marquée par le décès in utéro.

## Conclusion

Le syndrome miroir est une entité pathologique exceptionnelle dont le mécanisme physiopathologique demeure mal élucidé. Le diagnostic devrait être évoqué devant l´association d´une prééclampsie maternelle à une anasarque fœtale. La prise en charge doit être précoce devant la sévérité du pronostic materno-fœtal. Un traitement spécifique in utéro est vivement souhaitable car il permet la régression des anomalies clinico-biologiques materno-fœtales. Quoique dans certains cas graves idiopathiques, seule l´obtention de la vacuité utérine permet l´amélioration du pronostic maternel voire fœtale.
